# Supplementary material for: Evidence for Dosage Compensation in Coccinia grandis, a Plant with a Highly Heteromorphic XY System
Source: Genes (Basel). 2020 Jul 13;11(7):787. doi: 10.3390/genes11070787 (PMC7397054; doi:10.3390/genes11070787)

genes

MDPI

Supplementary Material

Supplementary Table 1. Library sizes and statistics for RNA sequencing from *C. grandis* male and female flower buds. Data characteristics before and after filtering out for sequencing adapters and low quality.

	**Total number reads**	**GC (%)**	**Total bases**	**Duplicates (%)**	**Total number reads**	**GC (%)**	**Total bases**	**Duplicates (%)**
C. grandis 1 – father	71,230,290	45.55	5,413,502,040	24.85	62,168,386	45.36	4,724,687,045	19.93
C. grandis 2 – mother	81,477,288	43.86	6,192,273,888	25.94	67,354,070	43.55	5,118,780,884	19.20
C. grandis 3 – son	74,106,838	42.40	5,632,119,688	39.80	53,535,608	42.02	4,068,592,348	30.21
C. grandis 4 – son	85,096,602	42.67	6,467,341,752	40.49	58,127,652	42.34	4,417,567,083	30.05
C. grandis 5 – son	76,653,508	43.63	5,825,666,608	37.12	56,078,580	43.14	4,261,849,202	27.72
C. grandis 6 – son	77,798,734	44.55	5,912,703,784	35.52	58,797,412	44.19	4,468,480,916	26.74
C. grandis 7 – son	72,948,152	43.55	5,544,059,552	36.73	55,608,456	43.27	4,226,126,310	27.83
C. grandis 8 – daughter	95,001,666	45.20	7,220,126,616	25.83	79,158,198	44.69	6,015,873,957	18.45
C. grandis 9 – daughter	76,074,360	44.79	5,781,651,360	20.00	67,478,518	44.44	5,128,249,651	14.98
C. grandis 10 – daughter	83,484,940	44.85	6,344,855,440	20.44	73,348,162	44.50	5,574,328,675	15.09
C. grandis 11 – daughter	77,883,860	44.74	5,919,173,360	20.94	73,348,162	44.36	5,194,162,562	15.44
C. grandis 12 – daughter	85,273,198	44.66	6,480,763,048	18.62	75,439,724	44.42	5,733,285,393	14.08
Total	957,029,436	44.20	72,734,237,136	28.86	780,442,928	43.86	58,931,984,026	21.64

Supplementary Table 2: BUSCO results for *C. grandis* flower bud transcriptomes. Assessment of transcriptome assembly compaleteness based on gene content from near-universal single copy orthologs. Contents of the final Trinity transcriptome and the working transcriptome containing the longest ORF predicted per Trinity isoform.

	Full Transcriptome	%	Longest ORF per isoform	%
Complete	1,115	77.43	1,033	71.74
Complete and single-copy	530	36.81	557	38.68
Complete and duplicated	585	40.63	476	33.06
Fragmented	176	12.22	193	13.40
Missing	149	10.35	214	14.86
Total groups searched	1,440		1,440	

Supplementary Table 3: Mapping statistics of all the samples. Number of reads from the *C. grandis* individuals mapping to the reduced trancriptome (the longest ORF predicted per Trinity isoform) with BWA and with GSNAP, a SNP-tolerant mapper. The male offspring libraries had higher PCR duplication rates than the female libraries (35.52-0.49% and 18.62-25.83% of raw reads respectively, see Supplementary Table 1) resulting in lower mapping rates (36.49 compared to 49.42% with SNP-tolerant mapping).

		BWA		GSNAP	
	#reads	#mapped reads	% mapping	#mapped reads	% mapping
C. grandis 1 – father	71,230,290	38,454,717	53.99	41,111,554	57.72
C. grandis 2 – mother	81,477,288	34,954,495	42.90	37,572,482	46.11
C. grandis 3 – son	74,106,838	24,609,806	33.21	27,041,875	36.49
C. grandis 4 – son	85,096,602	31,121,997	36.57	33,923,437	39.86
C. grandis 5 – son	76,653,508	29,660,482	38.69	32,261,535	42.09
C. grandis 6 – son	77,798,734	35,416,940	45.52	38,445,259	49.42
C. grandis 7 – son	72,948,152	30,938,500	42.41	33,763,165	46.28
C. grandis 8 – daughter	95,001,666	45,792,447	48.20	49,016,666	51.60
C. grandis 9 – daughter	76,074,360	38,958,015	51.21	41,562,663	54.63
C. grandis 10 – daughter	83,484,940	43,255,348	51.81	46,118,479	55.24
C. grandis 11 – daughter	77,883,860	39,070,638	50.17	41,756,525	53.61
C. grandis 12 – daughter	85,273,198	45,684,988	53.57	48,644,666	57.05
Total	957,029,436	437,918,373	45.76	471,218,306	49.24

Supplementary Table 4: Number of Differentially Expressed Genes (DEG) per gene categories.

	All		Autosomal		Sex-linked		X/Y		X-hemizygous	
	# contigs	%	# contigs	%	# contigs	%	# contigs	%	# contigs	%
All	82,699		3,706		1,364		1,196		168	
Differentially expressed	3,273	3.96	206	5.56	241	17.67	228	19.06	13	7.74
Male-biased	2,682	3.24	165	4.45	183	13.42	181	15.13	2	1.19
Female-biased	591	0.71	41	1.11	58	4.25	47	3.93	11	6.55

Supplementary Figure 1. Distribution of pairwise synonymous divergence (dS) between X and Y alleles in X/Y contigs. The vertical line indicates contigs longer than 1kb.

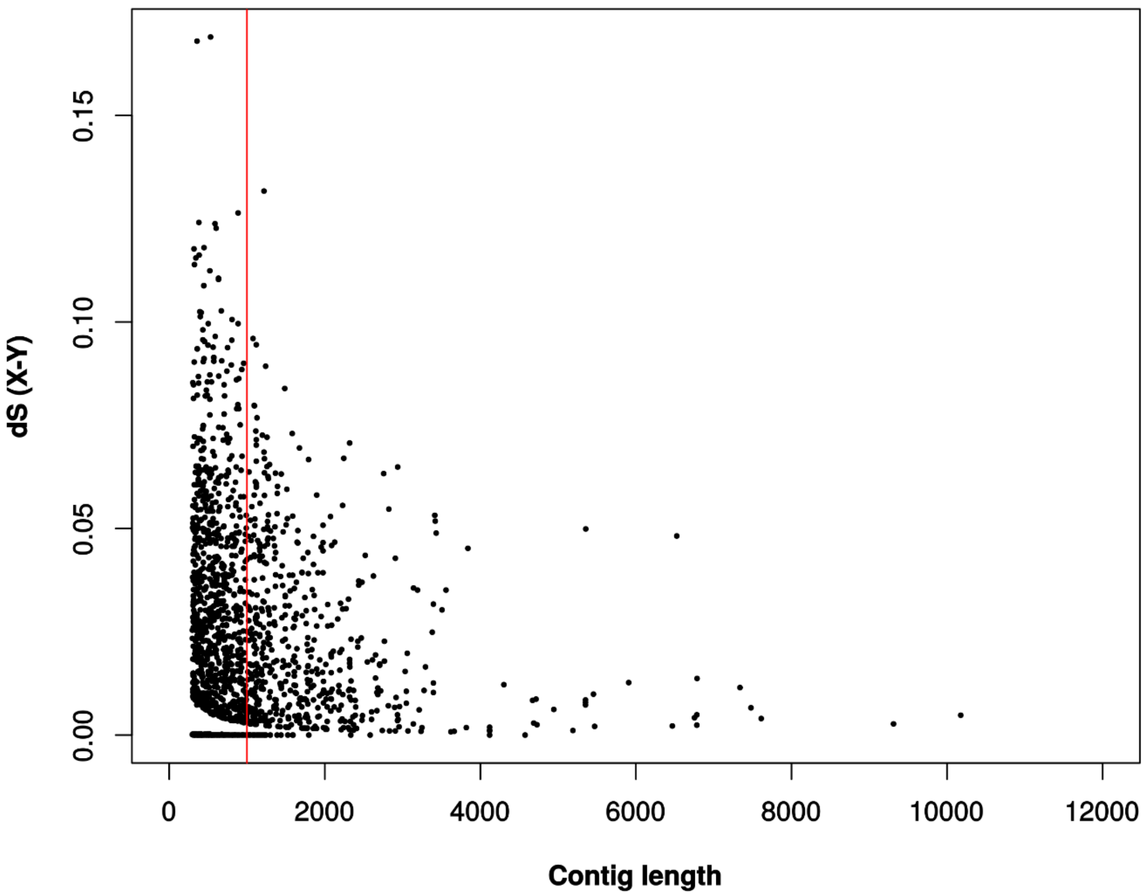

Supplementary Figure 2. Expression levels of autosomal and sex-linked contigs in both sexes before and after correction. Total read numbers were summed at SNP locations in each autosomal (A and C) and sex-linked (B and D) contig and normalized for each individual separately; medians for all individuals of the same sex were then obtained. Expression was corrected (C and D) using the autosomal median male/female ratio.

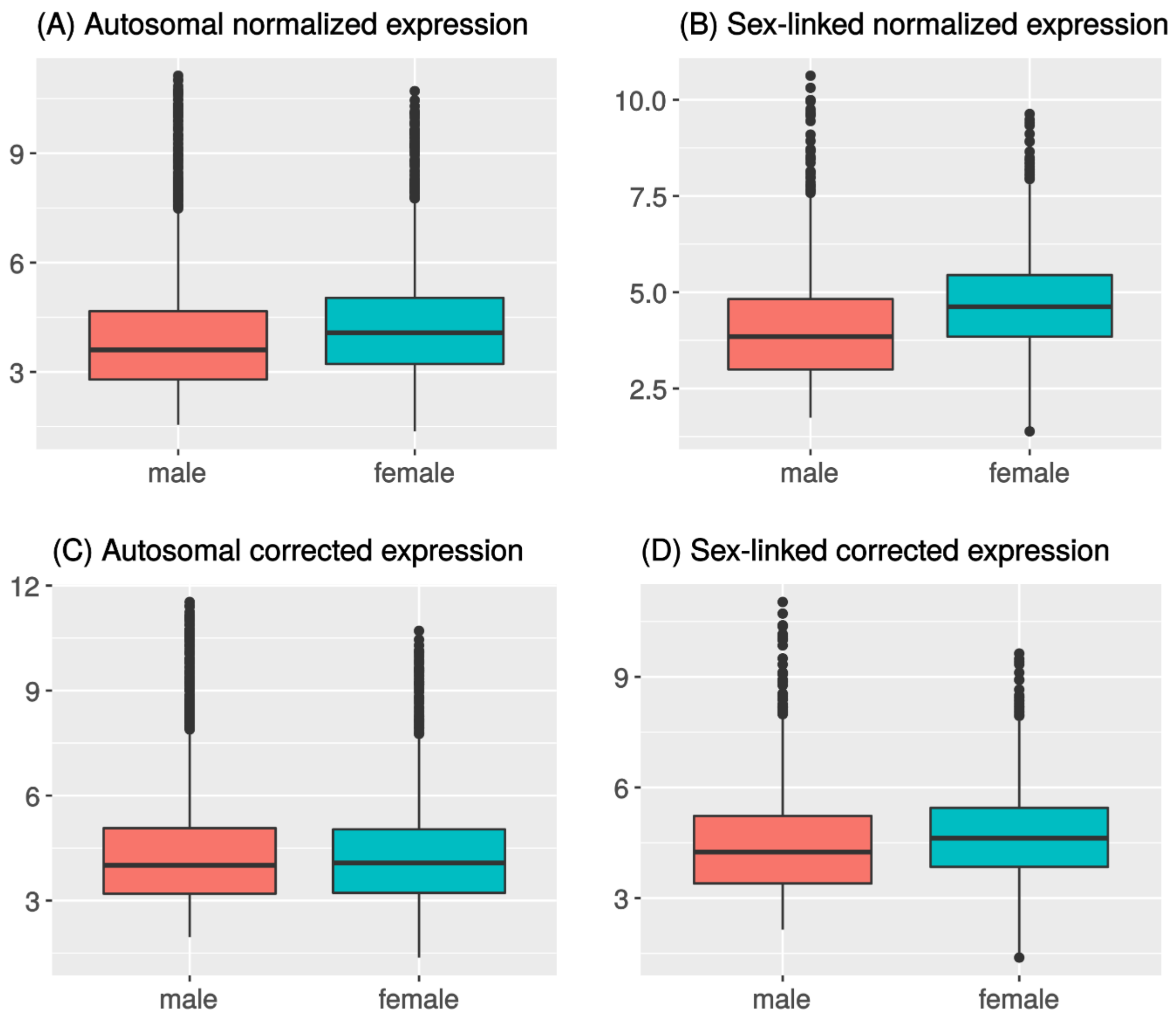

Supplementary Figure 3. Male vs. female autosomal gene expression ratio after correction. Distribution of normalized expression difference between males and females in autosomal contigs. Total read numbers were summed at autosomal SNP locations for each contig and normalized for each individual separately, then averaged across sexes to obtain the male/female ratio. Expression was corrected using the autosomal median male/female ratio. Distribution is shown in log_2 scale with its density curve. The median is shown in red.

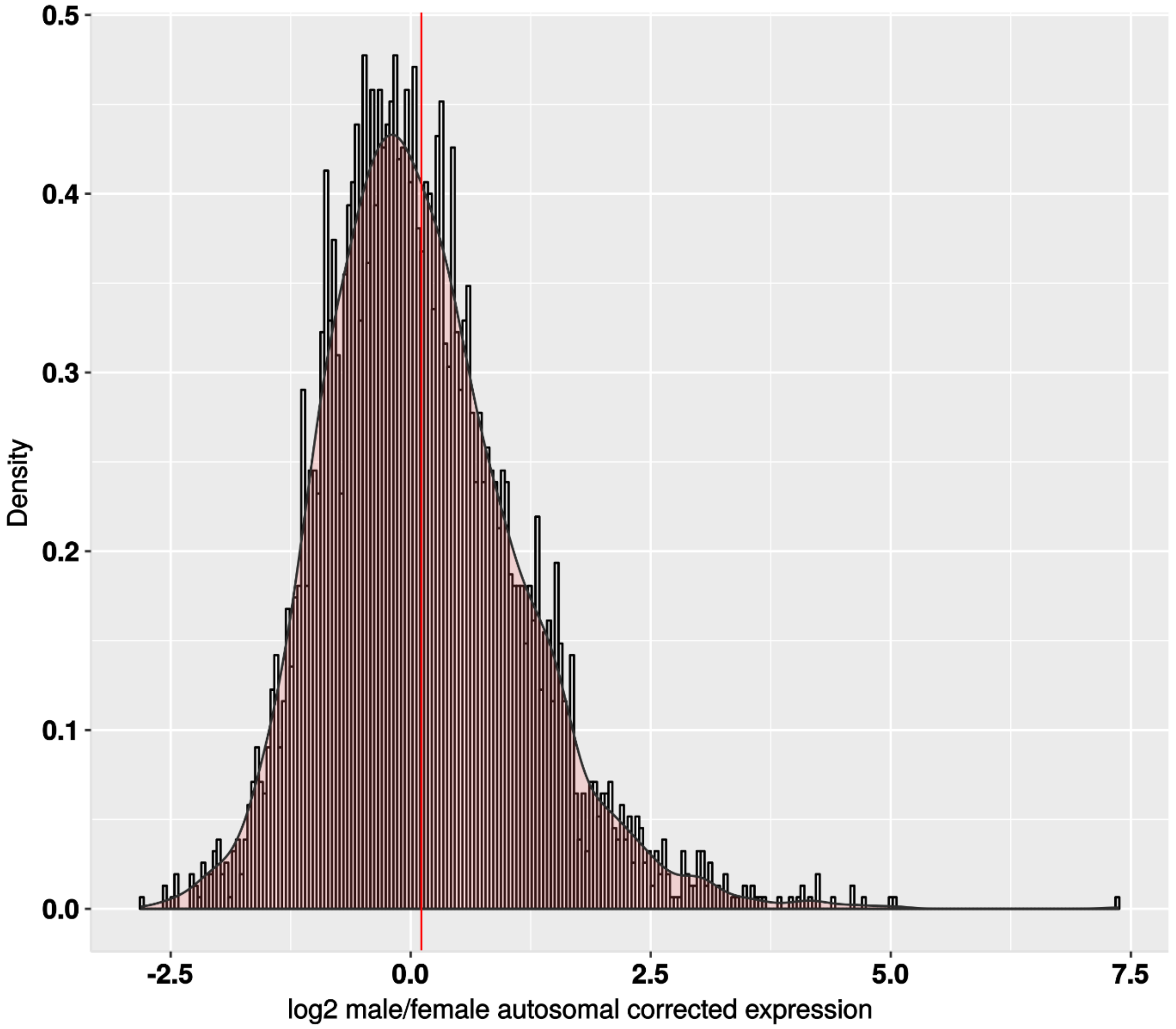

Supplementary Figure 4. Male vs. female sex-linked gene expression ratio after correction. Distribution of normalized expression difference between males and females in sex-linked contigs. Total read numbers were summed at sex-linked SNP locations for each contig and normalized for each individual separately, then averaged across sexes to obtain the male/female ratio. Expression was corrected using the autosomal median male/female ratio. Distribution is shown in log_2 scale with its density curve. The median is shown in red.

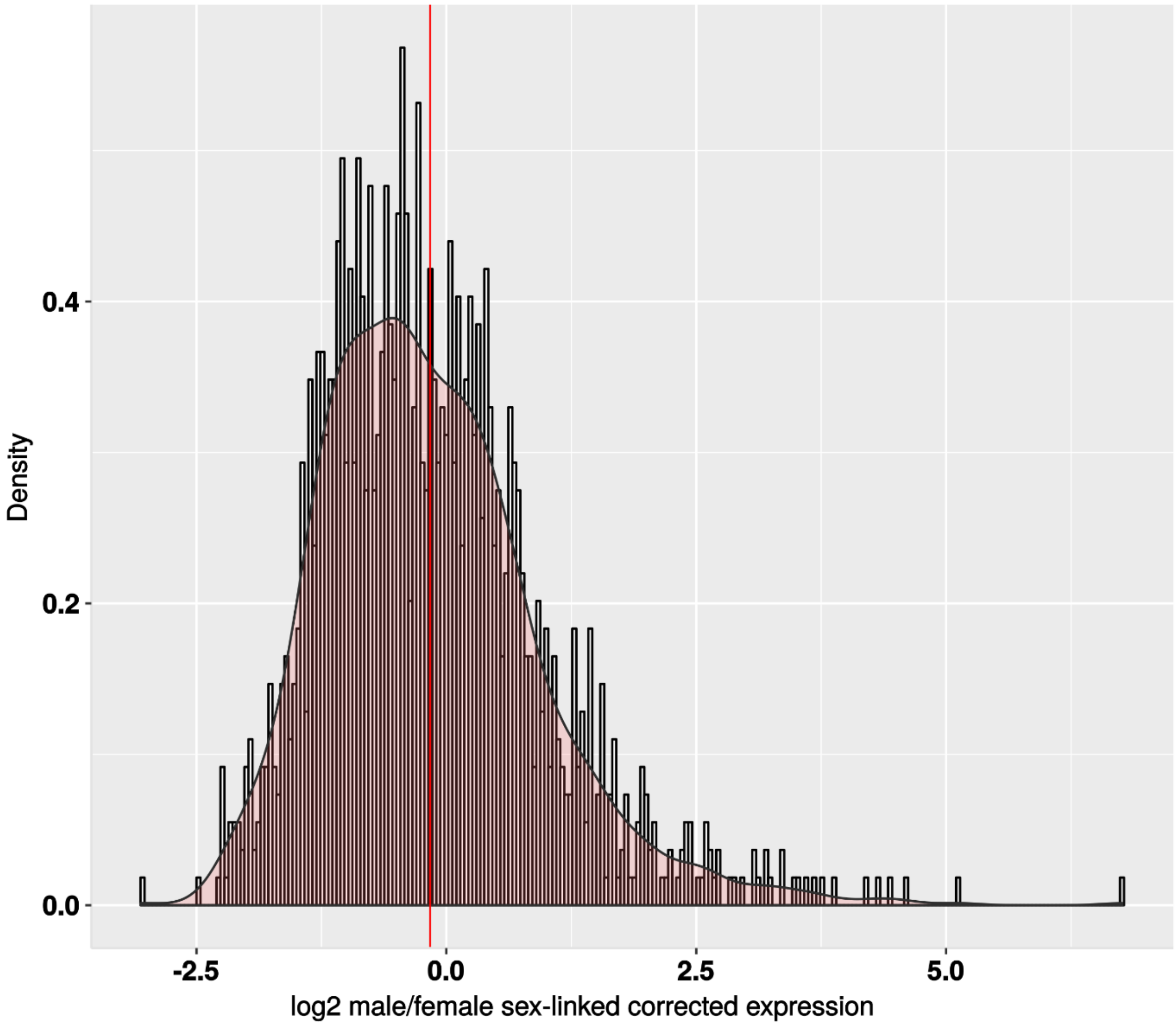

Supplementary Figure 5. Venn diagram of Differentially Expressed Genes identified by DESeq2, edgeR, and LimmaVoom. Number of genes with FDR <0.0001 for each method. 3,273 differentially expressed genes were identified by at least 2 methods.

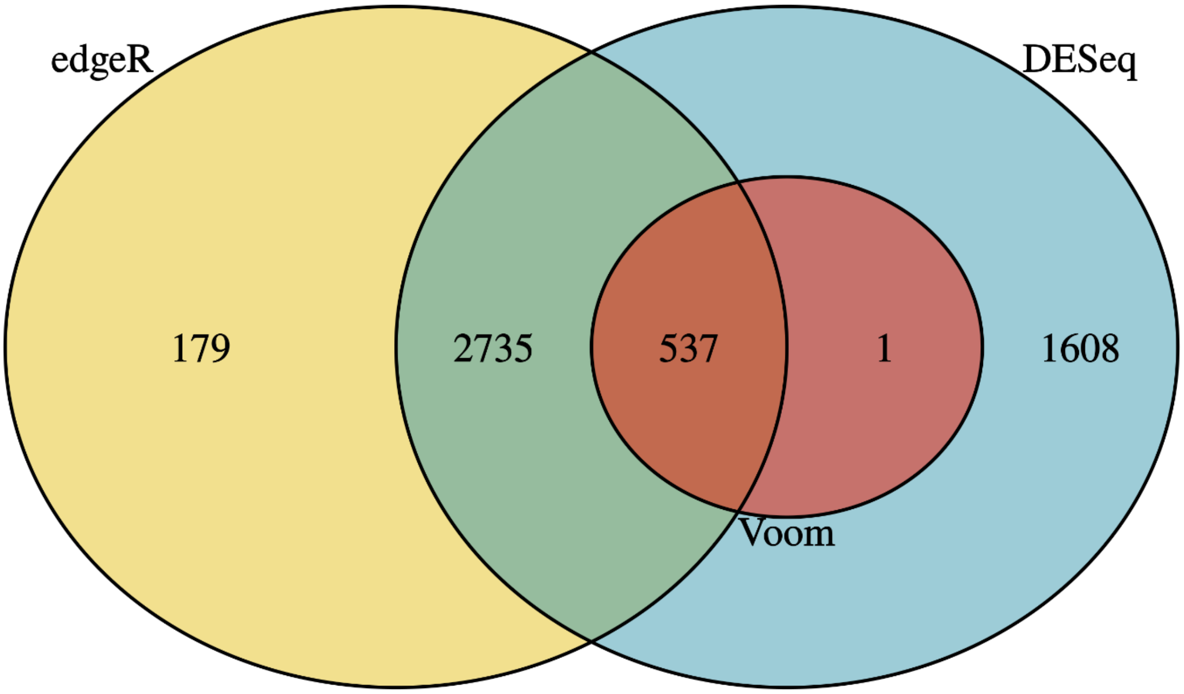

Supplement: Supplementary file 1 [file genes-11-00787-s001.pdf]
